# Foreign educated nurses’ work experiences and patient safety—A systematic review of qualitative studies

**DOI:** 10.1002/nop2.146

**Published:** 2018-04-17

**Authors:** Berit Viken, Eva Merethe Solum, Anne Lyberg

**Affiliations:** ^1^ Centre for Women's, Family and Child Health Faculty of Health and Social Sciences University College of Southeast Norway Kongsberg Norway

**Keywords:** foreign educated nurse, patient safety, transition, work experience

## Abstract

**Aim:**

The aim of this systematic review was to identify the evidence contributed by qualitative research studies of foreign educated nurses’ work experiences in a new country and to link the results to patient safety competencies.

**Design:**

A systematic literature review of qualitative studies.

**Methods:**

Electronic searches in the Ovid MEDLINE, Embase, PsycINFO, Cochrane Library and Cinahl databases and additional manual searches in five scientific journals. A content analysis of 17 qualitative articles was conducted.

**Results:**

The analysis revealed one main theme: “Being an outsider at work” and two themes: “Cultural dissonance and Unfamiliar nursing practice. Two sub‐themes emerged from the first theme; Loneliness and discrimination” and “Communication barriers”. The second theme was based on the following two sub‐themes: “Handling work‐related stress” and “Role uncertainty and difficulties in decision‐making”. A better prepared and longer orientation period with continual clinical supervision including systematic reflection on practice experiences is needed to support foreign educated nurses in the transition period and strengthen their Patient Safety Competencies. Nurse Managers have an important role in ensuring the inclusion of foreign educated nurses and providing desirable working conditions.

## 1 INTRODUCTION

The migration of nurses due to nursing shortages has been considered a global concern (An, Cha, Moon, Ruggiero, & Jang, 2016). In 2013, the International Council for Nurses Workforce Forum found that most industrialized countries were or would be facing a shortage of nurses due to increased demands for health care (Li, Nie, & Li, 2014). In this review, the term foreign educated nurses (FENs) refers to nurses who were born, raised and educated in another country to that where they work. FENs constitute a significant proportion of the nursing workforce in many Western countries (Bae, 2012; Cutcliffe, Bajkay, Forster, Small, & Travale, 2011). The healthcare sector has often relied on FENs to fill nursing vacancies (Larsen, 2007; Victorino Beechinor & Fitzpatrick, 2008). There has been a rise in the number of FENs in hospitals and especially in the long‐term care of the elderly and home‐care facilities (Brush, 2008).

### 1.1 Background

The World Health Organization (WHO) has proposed a Global Code of Practice on the International Recruitment of Health Personnel. It contends that if recruitment is properly managed, the international migration of health personnel can make a sound contribution to developing and strengthening health systems (WHO, 2010). The WHO emphasizes that all migrant health personnel should be offered appropriate orientation programmes that enable them to operate safely and effectively in the health system of the country where they work.

Recruitment ethics is a central issue, also in terms of safeguarding the rights of health personnel. The International Council of Nursing (ICN) Position Statement on Scope of Nursing Practice (ICN, 2013) states that employers have a responsibility to support nurses in practicing within their full scope of practice. This includes not placing nurses in situations where they are asked to practice beyond their level of competence or outside their legal scope of practice and providing practice environments that support safe and competent care.

Jeon and Chenoweth (2007) point to the many unresolved issues associated with the employment of FENs worldwide, such as treatment and rights in recruitment processes, equal opportunity in the workplace and the challenges and experiences of FENs, particularly during the transition period. FENs bring a wealth of experience, knowledge, skills and many personal attributes to their new country, but migration is not without challenges for the nurses themselves. They have to face a period of integration and adaptation as well as continual professional development (Hancock, 2008).

Nurses migrate not only for better pay, but also for an improved quality of life and better working conditions or opportunities for professional development, (Nichols, Davis, & Richardson, 2011). According to nursing researchers (Batnitzky & McDowell, 2011; Lum, Dowedoff, Bradley, Kerekes, & Valeo, 2015), very little academic attention has been paid to the challenges faced by FENs in their working lives. However, there is a growing body of literature pertaining to FENs (Okougha & Tilki, 2010). Research from the UK shows that FENs are often subjected to stereotypical and normative assumptions about their attributes and skills from colleagues, managers and patients that undermine their self‐confidence and may cause them to suffer stress (Alexis, Vydelingum, & Robbins, 2007; Allan, Cowie, & Smith, 2009).

With an increasingly diverse workforce, there is a need for healthcare organizations to address the challenges of skill transfer, role definition and communication. Ensuring safety is the cornerstone of the credentialing process for FENs, but nursing licensure requirements vary between countries (Sherwood & Shaffer, 2014; Sochan & Singh, 2007; Xu & He, 2012). For example, FENs in Canada reported that the credentialing process was inefficient, time‐consuming and expensive (Sochan & Singh, 2007). In Europe, the European Economic Agreement (EEA) provides for the free movement of persons, goods, services and capital in the European Single Market. The EEA member states have established mutual agreements that govern the recognition of professional qualifications (van Riemsdijk, 2010).

The Commission of European Communities (2008) defined patient safety as the prevention of unnecessary or potential harm associated with health care. Other definitions are related to the dynamic system of health care and focus on the interaction of several elements (WHO 2008; WHO, 2010). These definitions assume that incidents are the result of ineffective interaction between the actors involved (Wiig & Lindøe, 2009). Patient safety requires qualified and committed nursing staff competent in skills and effective in communication (Nease, 2009).

Patient safety culture is a subset of organizational culture specifically relating to the values and beliefs associated with patient safety (Feng, Bobay, & Weiss, 2008). Mustard (2002) defines patient safety culture as “a product of social learning, ways of thinking and behaving that are shared and that work to meet the primary objective of patient safety” (Mustard, 2002, p. 112). Nurses are in a key position to improve the quality of health care through patient safety interventions and strategies (Mitchell, 2008). A large body of literature on evidence‐based improvement strategies has been developed to enhance the quality of care and strengthen safety culture. Habermann and Stagge (2010) explored the impact of FENs on nursing care and professional standards. They concluded that the recruitment of FENs has not yet taken quality and safety issues and indicators in healthcare settings into consideration to the necessary extent.

In the context of preventing harm attention to non‐technical skills is crucial, defined as an array of cognitive, social and personal resource skills that compliment technical skills (Flin, O'Connor, & Crichton, 2008). According to The Canadian Patient Safety Institute, non**‐**technical skills comprise six core competency domains that reflect the knowledge, skills and attitudes that enhance patient safety across the continuum of care (Frank & Brien, 2008). The domains are: “Contribute to a culture of patient safety, work in teams for patient safety, communicate effectively for patient safety, manage safety risks, optimize human environmental factors and recognize, respond to and disclose adverse events.” (Frank & Brien, 2008, p. 4).

Examples of non‐technical skills often include being able to understand the situation, decision‐making, professional performance, interpersonal, leadership, team work and ethical skills (Mitchell et al., 2011; Yule, Flin, Paterson‐Brown, & Maran, 2006). It is estimated that between 70% and 80% of healthcare errors can be attributed to a breakdown in these skills (Flin et al., 2008).

The aim of this systematic review was to identify the evidence contributed by qualitative research studies of FENs’ work experiences in a new country and to link the results to patient safety competencies. The review question addressed was: What are FENs’ experiences and in what way can these experiences affect patient safety?

## 2 DESIGN

A systematic review was performed (Dixon‐Woods, Agarwal, Jones, Young, & Sutton, 2005; Thomas & Harden, 2008) on qualitative evidence to identify peer‐reviewed articles on the topic of interest. There is a continuum of processes and techniques between traditional literature reviews and systematic reviews. Systematic reviews involve a protocol, search, appraisal and synthesis, making them more likely to be rigorous and unbiased than a traditional review (Pawson & Bellamy, 2006).

## 3 METHOD

The search strategies were designed in collaboration with a specialized librarian. The inclusion criteria were: Peer‐reviewed articles in the English language based on original empirical studies. The articles should focus on the FENs**’** perspective on their work experiences. After the initial search, the criteria were limited to qualitative articles published after 2005. The following search words were selected: Foreign, immigrant, ethnic, international, multicultural**,** cross‐cultural, abroad, nurse, nursing, cultural competency, cultural diversity, cultural sensitivity and patient safety. Electronic searches were conducted in the Ovid MEDLINE, Embase, PsycINFO, Cochrane Library and Cinahl databases and resulted in a total of 1,054 matches. This was reduced to 116 after reading the titles. Further reduction took place after all three authors had read the abstracts. Reasons for excluding articles were reviews, the absence of primary data and quantitative methods. Following closer examination of 43 articles, 38 were excluded and five were selected for further analysis. The reason for excluding these articles was that they did not focus on FENs’ perspective. Further manual searches were conducted in Swemed and in five scientific journals, resulting in the inclusion of an additional 11 articles.

**Figure 1:**
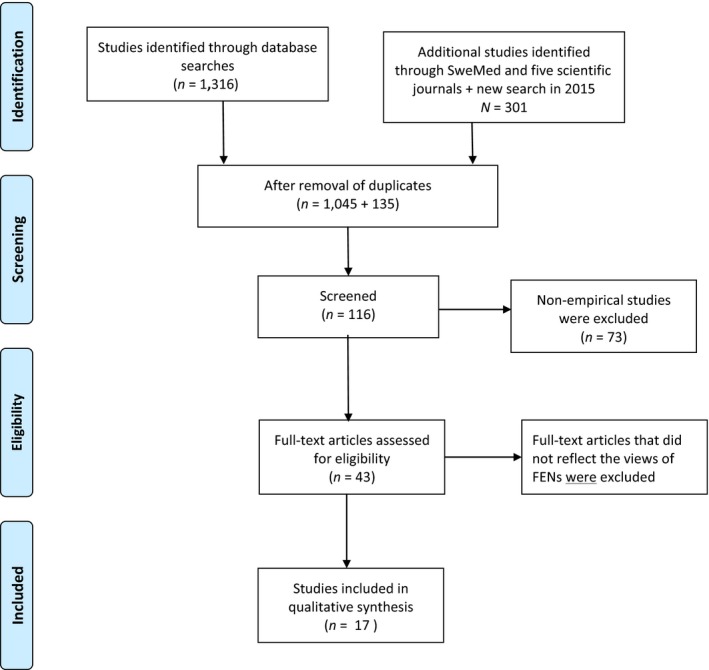
Flow diagram

Through the process of reading, the authors were able to select more specific search words and an updated search was performed in December 2016 in the same databases, plus Web of Science and ProQuest. The search words were: Foreign or international, nurse, job or work experience. Seven articles were found, of which six were duplicates from the first search, thus one new article was included (Smith, Fisher, & Mercer, 2011). This gave a total of 17 articles for review (Figure 1).

### 3.1 Quality appraisal of the included articles

The methodological quality of the included studies was assessed and rated according to the Critical Appraisal Skills Program (CASP), a methodological checklist of key criteria relevant to qualitative studies (CASP, 2013). The three authors (BV, EMS, AL) assessed the quality of the studies together. Consensus was achieved by discussing the studies in the light of the various criteria. It was finally agreed that six studies were of moderate methodological quality, while 11 studies had high methodological quality (Appendix 1). No studies were excluded due to low quality.

### 3.2 Analysis and synthesis of the included studies

The included studies employed different qualitative approaches such as individual interviews, reflective diaries, world café, focus groups, narratives from guided interviews and a single‐case study. A thematic analysis was performed, which includes different steps (Thomas & Harden, 2008). In the first step, the authors individually read the studies using free line by line coding, followed by discussions to achieve consensus and strengthen validity. The second step involved organizing the codes into descriptive themes. The authors agreed on a classification system for sorting and analysing the content of the articles at a descriptive level. They spent time discussing and comparing the different types of evidence as a further step to a synthesis of the FENs’ work experiences. The synthesis was finally achieved when the authors reflected on the content, abstracted it and after a discussion agreed on the theoretical themes (Thomas & Harden, 2008). A new interpretation, which goes beyond the original studies was formulated**.**

**Table 1:** List of included qualitative studies, their characteristics and contributions to the results

Author, year, country	Aim/objective	Methods and study setting	Data analysis	Results	Quality score
1. Almutairi et al. (2015) Saudi Arabia	To explore notions of cultural competence with non‐Saudi Arabian nurses working in a major hospital in Saudi Arabia	A single‐case study design in an 800‐bed teaching hospital. Face‐to‐face audio‐recorded, semi‐structured interviews with 24 non‐Saudi Arabian nurses from North America, Africa, Europe, Asia, Australia, New Zealand and the Middle East	Deductive analysis according to Campinha‐Bacote's framework for cultural competence (Campinha‐Bacote, 2002) and inductive analysis	Nurses within this culturally diverse environment struggled with the notion of cultural competence in terms of each other's cultural expectations and those of the dominant Saudi culture The findings emphasize how cultural skills within a multicultural context are also related to the nurse's ability to integrate and apply cultural knowledge that is not confined to interactions with patients; it is also dependent on skilful interactions with other healthcare professionals to ensure safe and effective care Organizational support might be implemented to help nurses overcome the dissonance and disempowerment they often encounter	High
2. Connor and Miller (2014) USA	To identify the perceived sources of work‐related and non‐work‐related stress and describe the factors that influence stress in Filipino immigrant nurses	A cross‐sectional qualitative design. 20 Filipino women working as registered nurses in the Chicago metropolitan area were interviewed	Auerbach and Silverstein's method (Auerbach & Silverstein, 2003)	The immigrant nurses’ stress was influenced by both the situational and cultural context, which contributed to the complexity and multidimensionality of their experience. Understanding the specific stressors faced by immigrant nurses will assist in developing/will facilitate the development of culturally appropriate interventions to mitigate the nurses’ stress and challenges	High
3. Estacio and Saidy‐Khan (2014) UK	To explore the experiences of racial microaggression among migrant nurses in The United Kingdom	Eleven migrant nurses in two hospitals in the Midlands of England, nine originally from the Philippines, one from Kenya, one from Zimbabwe and one from Zambia. The nurses kept a reflective diary for 6 weeks to record and reflect on their experiences of living and working in The United Kingdom. They were contacted by phone every 2 weeks. A World Café event was organized to bring all the participants together.	Transcripts of the diaries were thematically analysed (Braun & Clarke, 2006)	The participants’ diaries reflected incidences of racial microaggression. At the micro level, migrant nurses experienced racial microaggression through racial preferences and bullying. Institutional racism also hindered the nurses’ opportunities for further training and promotion. Organizational infrastructures need to be in place to encourage better multicultural interactions in the workplace	Moderate
4. Kishi et al. (2014) Australia	To investigate the experiences of Japanese nurses and their adaptation to their work environment in Australia	Individual semi‐structured interviews with 14 Japanese registered nurses working in Australian hospitals	Thematic analysis was used for identifying, analysing and reporting patterns (themes) within the data (Braun & Clarke, 2006)	There is a need to better support overseas qualified nurses (OQNs) as they progress to a stage of settling as registered nurses in a new country. Workplace mentorship programs tailored to the needs of OQNs and based on a greater understanding of those needs may ease their transition towards becoming more effective members of the healthcare team. Education of nursing colleagues about the personal experiences of OQNs may help to build more empathic relationships between locally qualified nurses and OQNs	High
5. Xiao et al. (2014) Australia	To examine relationships between social structures and nurses’ actions that either enabled or inhibited workforce integration in hospital settings	Focus groups and face‐to‐face in‐depth interviews with 24 immigrant nurses from China, Columbia, Malaysia, Singapore, South Korea and Zimbabwe with non‐English‐speaking backgrounds. Many had more than 10 years nursing experience in their home country	Structuration theory (Giddens, 1984) with double hermeneutic methodology was used to interpret the nurses’ perceptions of factors affecting workforce integration	A multicultural team places stress on both host nurses and immigrant nurses as they need to learn from each other and make allowances for team members. Organizational policies and procedures can also generate discrimination that impact on migrant nurses. A regular review of the organization's policies and procedures to reflect the ever‐changing workforce is imperative	High
6. Zhou (2014) Australia	To explore experiences of China educated nurses working in Australia	46 in‐depth interviews with 28 China‐educated nurses at two sites	Based on grounded theory using the constant comparison method (Charmaz, 2006)	Unrealistic expectations predisposed the nurses to many hardships, disappointments and frustrations. Providing access to adequate and realistic information that ensures a balanced view of immigration life would be beneficial. More dialogue and discussion are needed to promote mutual understanding and increase the efficacy of support services	High
7. Alexis (2013) UK	To gain an understanding of international educated nurses’ (IENs) experiences of working in the National Health Services in England	A hermeneutic phenomenology approach with two phases: Phase 1: 12 individual interviews with IENs in a District General Hospital in England. Phase 2: 4 focus group interviews. The informants originated from The Philippines, South Africa, The Caribbean and Sub‐Saharan Africa	The data were thematically analysed using a qualitative data analysis software package and guided by Van Manen's analytical framework (Manen, 1990)	IENs encountered a number of challenges to their work practices in an English hospital. There is a need for both IENs and home ‐grown nurses to adapt to each other's cultural differences. Support is necessary as it leads to job satisfaction with the ultimate effect of better patient care	High
8. Lin (2013) USA	How Filipina nurses transition into and adapt to nursing practice in the US Hospital system	In‐depth interviews with 31 Filipina nurses employed in a hospital setting in Texas	Grounded theory (Strauss & Corbin, 1998)	Nurses from other countries can encounter hardships associated with cultural dissimilarities, language barriers, diversity in nursing practice, differences in social norms and feelings of alienation	Moderate
9. Wheeler et al. (2013) USA.	To gain a deeper understanding about the experiences of internationally educated nurses (IENs) compared to US registered nurses (RNs) practicing in two urban hospitals in south eastern USA	A cross‐sectional, qualitative descriptive design. Two phases of semi‐structured interviews. The second phase comprised a more in‐depth interview with 9 US RNs and 11 IENs from Oceania, the Pacific, East Asia, Southwest Asia, Africa (Sub‐Sahara), the European Union and the Caribbean	The constant comparative method (Creswell, 2003)	IENs and newly licensed US RNs faced similar barriers when they began practicing in the USA, but IENs faced additional challenges of adjusting to the attitudes of US patients, the perceived lack of respect for nurses and delivering total patient care IENs would benefit from orientation regarding/in terms of the cultural differences in the USA. Healthcare facilities might consider extra orientation for IENs to help them understand their new environment and perform to the best of their abilities	Moderate
10. Alexis and Shillingford (2011) UK	To explore the perceptions and work experiences of internationally recruited neonatal nurses (IRNNs)	Husserl’s phenomenological approach underpinned the study. Semi‐structured face‐to‐face interviews with 13 nurses	Colaizzi's analytical framework was used to analyse the data (Colaizzi, 1978)	Given that IRNNs are part of the workforce, it is important to offer them support as this could enable them to provide high quality care Accepting, valuing and supporting IRNNs can create a much more satisfying workforce and ultimately improve the standard of care for patients. This study highlights the inconsistencies in the nature of support for IRNNs in that only some participants received support from colleagues as well from their managers, which enabled them to become far more confident in their work environment	High
11. Jose (2010) USA	To elicit and describe the lived experience of internationally educated nurses (IENs) who work in a multihospital medical centre in the urban USA	A phenomenology of practice design. Narratives from guided interviews with 20 new IENs from the Philippines, Nigeria and India were collected	Giorgi's principle of data analysis (Giorgi, 1985)	Inadequate orientation was a predominant concern voiced by the participants. Due to the paucity of research about US IENs, there is a possibility that the hospitals were unable to identify the learning outcomes and provide appropriate content suited to the needs of IENs. Building on the IENs’ solid education from their homelands and their individual strengths were emphasized	High
12. Liou and Cheng (2011) USA.	To explore and interpret the lived experience of a Taiwanese nurse working in a U.S. Hospital	A single‐case study. Four in‐depth telephone interviews were conducted	The interpretive process of the hermeneutic circle was used to avoid inappropriate interpretations and to produce rich descriptions and in‐depth interpretations (Cohen, Kahn & Steeves, 2000)	The nurse felt that due to the language barrier she was unable to provide the highest quality of nursing care of which she was capable in the United States. Support from people in her work environment was especially important for avoiding or repairing breakdowns in communication. Advantages of working in the U.S. nursing system include sick leave and a more humane patient–nurse ratio	High
13. Smith et al. (2011)	To explore how overseas qualified nurses (OQNs) experienced the practice of nursing in Australia	Individual interviews with 13 nurses from nine different countries; China, South Africa, Japan, Taiwan, Zimbabwe, Hong Kong, The Philippines, Sweden and Nepal	Moustakas’(1994) scientific approach to phenomenological analysis	A major issue was holistic patient care as the participants had not attended to basic nursing care in their previous practice. Lack of communication and problem‐solving skills, in addition to unfamiliarity with documentation and patient education made them feel disempowered	High
14. Allan (2010) UK	To examine the experiences of mentoring for overseas‐trained nurses in the UK	An ethnographic interpretative study using mixed methods. Results reported in this article are based on individual in‐depth interviews with 93 overseas‐trained nurses	Qualitative analysis	The main barrier to effective and non‐discriminatory mentoring was the lack of preparation within both the NHS and the care home sector about how cultural differences affect mentoring and learning for overseas‐trained nurses. They were concerned that their existing skills were not up to British standards. They experienced the need to nurse in the “British” way, in addition to/as well as bullying and discriminatory practices in the workplace	Moderate
15. Tregunno et al. (2009) Canada	To gain greater insight into the degree to which internationally educated nurses (IENs) are competent to practice safely upon registration	Semi‐structured interviews of 30 nurses from 20 countries. Half of the participants worked in long‐term care, two in community and the remainder in acute care settings. Years in Canada: 3 (on average)	Constant comparative methods (Strauss & Corbin, 1998)	The IENs reported that nurses in Canada were expected to be more assertive, to have more responsibility for patients, to be more involved in decision‐making and to have more egalitarian relationships with physicians than they were accustomed to. The results highlight areas of potential public risk. “The novice to expert model” is used to understand the need for additional intervention and successful workplace transition	Moderate
16. Yu, Gutierrez, & Kim, (2008) USA	To examine Chinese nurses’ lived experiences in the US healthcare environment	9 in‐depth interviews with Chinese nurses working in the US conducted in English	Colaizzi's analytical framework (1978) was used to analyse the data	The study revealed real and potential risks for patient safety and quality of care. However, these nurses managed to turn challenges into opportunities: High level of job satisfaction, desire for learning, execution of strategic plans and career enhancement through further education, proactive in terms of adapting to workplace demands	High
17. Magnusdottir (2005) Iceland	To generate an understanding of the lived experience of foreign nurses working at hospitals in Iceland	The Vancouver school of doing phenomenology. Dialogues with 11 registered nurses from seven countries (nationalities not specified because of ethical issues)	Thematic analysis	The voices of foreign nurses in international data banks are predominantly the voices of English‐speaking nurses from developing countries who migrate for financial reasons and work in English‐speaking industrialized countries. The foreign nurses had stressful experiences as “the other”. Co‐workers and supervision may be more important than personal support. The language barrier was central to the nurses’ experience	

Auerbach, C.& Silverstein, L. B. (2003). *Qualitative data: An introduction to coding and analysis*: NYU press.

Braun, V. & Clarke, V. (2006). Using thematic analysis in psychology. *Qualitative research in psychology, 3*(2), 77‐101.

Campinha‐Bacote, J. (2002). The process of cultural competence in the delivery of healthcare services: A model of care. *Journal of Transcultural Nursing, 13*(3), 181‐184.

Charmaz, K. (2006). Constructing Grounded Theory: A Practical Guide through Qualitative Analysis (Introducing Qualitative Methods series).

Cohen, M., Kahn, D. & Steeves, R. (2000). *Hermeneutic Phenomenological Research: A Practical Guide for Nurse Researchers*: Sage publications.

Colaizzi, P. F. (1978). Psychological research as the phenomenologist views it. In R. S. Valle & M. King (Eds.), *Existential‐Phenomenological Alternatives for Psychology* (pp. 6): Oxford University Press.

Creswell, J. W. (2003). Research design: Qualitative, quantitative, and mixed methods design. *Sage, London*.

Giddens, A. (1984). *The constitution of society: Outline of the theory of structuration*: Univ of California Press.

Giorgi, A. (1985). Sketch of a psychological phenomenological method. *Phenomenology and Psychological Research. Duquesne University Press, Pittsburgh*, 8‐22.

Manen, M. V. (1990). Researching lived experience. *State University of New York Press, New York, NY*.

Moustakas, C. (1994). *Phenomenological Research Methods*: Sage publications.

Strauss, A. & Corbin, J. (1998). Basics of qualitative research: Procedures and techniques for developing grounded theory: Thousand Oaks, CA: Sage.

**Table 2:** Overview of the interpreted main theme, themes and sub‐themes

Main theme: Being an outsider at work	
Theme I: Cultural dissonance	Theme II: Unfamiliar nursing practice
Sub‐themes: Loneliness and discrimination Communication barriers	Sub‐themes: Work‐related stress Role uncertainty and difficulties in decision‐making

### 3.3 Ethics

Ethical approval was not required (Table 1).

## 4 RESULTS

The included studies covered FENs of many nationalities working in different countries such as the UK, USA, Iceland, Saudi Arabia and Australia. They mainly described experiences of nurses from Asia and Africa. Only a few of the articles dealt with the situation of nurses from European countries. Although the FENs’ experiences varied according to their country of origin and destination, there were nevertheless many similarities.

The analysis revealed one main theme: “Being an outsider at work” and two themes: “Cultural dissonance” and “Unfamiliar nursing practice”. Two sub‐themes emerged from the first theme: “Loneliness and discrimination” and “Communication barriers”. The second theme had the following two sub‐themes: “Handling work‐related stress” and “Role uncertainty and difficulties in decision‐making” (Table 2).

### 4.1 Main theme: Being an outsider at work

The main theme was identified as Being an outsider at work. Feelings of being an outsider meant not being accepted and recognized by peers as a valuable and contributing team member. The feeling of otherness and uncertainty due to the new nursing practice had a negative impact on FENs’ work performance (Tregunno, Peters, Campbell, & Gordon, 2009).

### 4.2 Theme I: Cultural dissonance

Cultural dissonance occurred when FENs met a new professional environment with a different culture. Recruiting agents had provided them with limited or no information concerning the cultural requirements of the host country (Allan, 2010). There was a tension between the FENs’ desire to hold on to their old selves and the need to conform to the new society and working conditions (Zhou, 2014). The experiences of cultural dissonance compelled FENs to reflect on their identity as cultural beings, leading to (un)learning or reaffirming who they are (Xu, Gutierrez, & Kim, 2008). Feelings of loneliness, discrimination and profound communication barriers are all part of the cultural dissonance.

#### 4.2.1 Loneliness and discrimination

Unexpected changes in FENs’ social and cultural environment created a feeling of loneliness and discrimination (Alexis, 2013; Allan, 2010; Almutairi, McCarthy, & Gardner, 2015; Connor & Miller, 2014; Jose, 2011; Xu et al., 2008). The nurses described “a sense of loss”, “being thrown into an unfamiliar world” and being situated as “the other” (Magnusdottir, 2005; Tregunno et al., 2009; Xu et al., 2008; Zhou, 2014). A social psychological distance between the FENs and their colleagues functioned as an invisible wall, resulting in feelings that “we are among but we are not in” (Zhou, 2014, p. 4). Some FENs said that they felt lonely in the workplace because they usually found the topics of conversation unfamiliar and uninteresting, in addition to the fact that they did not engage in the same social activities as their colleagues. It could also be difficult to balance family and work life because the FENs lacked the support they would have had in their home country (Connor & Miller, 2014).

Many FENs perceived being discriminated against and that the other nurses resented their being hired (Connor & Miller, 2014; Estacio & Saidy‐Khan, 2014). They perceived mistreatment, intimidation and a lack of respect from others throughout the settlement process (Allan, 2010; Lin, 2013; Wheeler, Foster, & Hepburn, 2013; Xu et al., 2008). A nurse reported being bullied by being assigned the “hardest” patients to “see if she could survive” (Connor & Miller, 2014, p. 509). FENs could also experience contemporary forms of racism that were considered covert, subtle and sometimes unintentional. Patients also exhibited this form of racial microaggression by declining services offered by FENs. Colleagues used exclusion to make their targets feel unwanted and excluded from the group (Estacio & Saidy‐Khan, 2014). Some FENs developed strategies to overcome discriminatory remarks such as remaining silent and not complaining (Allan, 2010).

When moving to a new country, many FENs hoped to further their education (Kishi, Inoue, Crookes, & Shorten, 2014). However, the reality was often quite contrary to what they expected. Institutional racism could hinder their opportunities for further training and promotion, for example the lack of clear rules and resources to accredit FENs’ expertise (Xiao, Willis, & Jeffers, 2014). Some nurses described being treated like students and felt devalued (Alexis & Shillingford, 2011).

Despite the difficulties and unmet support needs, FENs demonstrated great strength and resilience (Zhou, 2014). Being open‐minded and assertive helped them to cope with the challenges they faced and overcome loneliness and discrimination (Alexis & Shillingford, 2011).

#### 4.2.2 Communication barriers

The challenge of learning the language and overestimation of their language fluency made the nurses feel anxious and insecure because they considered that it adversely influenced the quality of care they could provide (Almutairi et al., 2015). Both lack of language proficiency and different communication styles could lead to difficulties forming interpersonal relationships and adjusting to new environments (Magnusdottir, 2005). Passing a language test did not guarantee successful communication in the workplace. Intercultural communication ability was acknowledged by FENs as the most challenging aspect of their work and it took them a long time to adapt their communication style to that of the host country and to understand all the nuances (Xiao et al., 2014). Many FENs expressed a desire to learn the patients’ culture and language and were committed to providing culturally appropriate and safe care.

New ways of interacting with colleagues could also be quite frustrating for the FENs. Nurses who were used to only receiving written orders from the doctors in their home countries found verbal and telephone orders distressing and frustrating (Jose, 2011; Liou & Cheng, 2011; Xu et al., 2008).

### 4.3 Theme II: Unfamiliar nursing practice

FENs experienced an unexpected unfamiliar nursing practice. Although nursing may be considered universal, many FENs were poorly prepared for a new nursing practice culture (Alexis, 2013; Kishi et al., 2014; Xiao et al., 2014). The transition created work‐related stress as well as role uncertainty and difficulties in decision‐making. FENs faced challenges adjusting to the healthcare providers and the healthcare system (Zhou, 2014). Some FENs stated that nursing principles were viewed similarly, but that nursing practice differed.

#### 4.3.1 Work‐related stress

Inadequate orientation before coming to the new country and inconsistent support at the workplace were common among FENs (Jose, 2011; Zhou, 2014). Some nurses described the immigration process as long and difficult and wished they had received more help from the recruiters (Jose, 2011). Other FENs felt a lack of support from hospital managers (Almutairi et al., 2015). In the USA, several FENs were concerned about litigation and could behave in a certain way to protect their nursing license (Wheeler et al., 2013).

The new work environment was experienced as very stressful (Kishi et al., 2014). Some described “shocking workplace realities”, including demanding patients, high‐tech equipment and expanded nursing responsibilities. As already stated, they could experience communication problems, discrimination and alienation, which interacted with intensified work‐related stressors (Connor & Miller, 2014).

Stressors also came from some peers who criticized the FENs and saw their difficulties as incompetence rather than inexperience with a new system (Jose, 2011). For example, FENs working in a neonatal department were unfamiliar with family‐centred care and the need to communicate and provide parents with information, which caused stress (Alexis & Shillingford, 2011). On the other hand, having a new emotional connection with their patients and the patients’ families could reduce the work‐related stress (Lin, 2013).

The multicultural work team placed additional stress on both host nurses and FENs (Xiao et al., 2014). Many FENs had little former exposure to other races and ethnicities (Connor & Miller, 2014). They were therefore challenged by working with people from diverse cultures**,** each with their own pattern of behaviour, value systems and beliefs (Almutairi et al., 2015). Many FENs obtained support from colleagues of the same nationality to handle the work‐related stress, thus helping each other to adjust to a new culture (Alexis, 2013; Alexis & Shillingford, 2011). Some also obtained support from other colleagues as well as from their managers, which enabled them to become more confident in their work environment and was of the utmost importance for coping with work‐related stress (Alexis & Shillingford, 2011; Jose, 2011; Liou & Cheng, 2011; Magnusdottir, 2005).

#### 4.3.2 Role uncertainty and difficulties making decisions

It has been reported that FENs may “take a u‐turn” from clinical expert to cultural novice when they enter practice in a new country (Tregunno et al., 2009). Some had agreed to work outside their area of expertise to obtain an employer‐sponsored visa (Xiao et al., 2014). Many FENs did not have the knowledge required to care for specific populations and had to learn about, for example elder care, in preparation for the national registration examinations (Tregunno et al., 2009).

Expectations of critical thinking presented challenges for the FENs (Xu et al., 2008). Practice expectations differed from their country of origin, as the nurses in their new country were often expected to be more assertive, assume greater responsibility for patients and be more involved in decision‐making (Tregunno et al., 2009). Nurses were also expected to have more egalitarian relationships with physicians, while the culture in their country of origin was more oriented towards nurses just following orders. The FENs could feel “overwhelmed” because they had to learn to provide total patient care for a certain number of patients while they were accustomed to working in a team of several nurses, who shared the responsibility and decision‐making (Wheeler et al., 2013). Other nurses felt uncomfortable at the beginning due to the informality and lack of hierarchy among colleagues. They believed that there was inadequate discipline and that the response to mistakes was too mild. Some concluded that the quality of care was affected by this easy‐going culture, which led to role uncertainty (Magnusdottir, 2005). For example, in their country of origin the activities of daily living were mainly performed by family members who were at the bedside, while in the new country these activities were the responsibility of the nursing staff. It was difficult for some FENs to accept washing, toileting and feeding as part of a nurse's role (Smith et al., 2011; Zhou, 2014).

They had to learn that patients were at the centre of health care and that patient satisfaction with health care was emphasized (Alexis & Shillingford, 2011; Lin, 2013; Tregunno et al., 2009). The FENs had to change both their practice and the power relationship between themselves and their patients. The consumer‐centred approach was highlighted and patient education assumed a prominent role in nursing practice (Smith et al., 2011; Tregunno et al., 2009).

The FENs had to learn to make decisions autonomously while providing direct patient care (Lin, 2013). They could be reluctant to make decisions because they expected no protection in the event of making an incorrect judgement. For some FENs, the desire to appear competent and not lose respect rendered it difficult to disclose lack of knowledge and seek support. They therefore concealed and underplayed their doubts and attempted to work as normally as possible (Zhou, 2014). In the later stages of their adaptation to their new surroundings, FENs began to interact and communicate confidently as professionals, for example with doctors (Lin, 2013).

## 5 DISCUSSION

The aim of this systematic review was to identify the evidence contributed by qualitative research studies of FENs’ work experiences in a new country and link the results to patient safety competencies. The main theme *Being an outsider at work* and two themes; *Cultural dissonance* and *Unfamiliar nursing practice* that emerged from the analysis revealed that the experience of being an outsider at work makes FENs uncertain in their new practice environment, which has an impact on patient safety.

### 5.1 Cultural dissonance

The theme *Cultural dissonance* involves the FENs’ self‐confidence, their way of being and how reactions from others affect their communication and teamwork. The theme contains two sub‐themes: *Loneliness and discrimination* and *Communication barriers*. The difference between FENs’ own cultural background and the culture in the new country creates a feeling of loneliness. In addition, they are exposed to discrimination from colleagues and patients, which can undermine their self‐confidence and professional effectiveness (Larsen, 2007). Kingma (2008) contends that discrimination and marginalization of FENs threaten patient safety and disrupt the cooperation dynamic required to advance the delivery of care. Healthcare professionals should be mindful that members of the healthcare team need support when responding to adverse events (Frank & Brien, 2008).

Teamwork and communication are two of the main patient safety competency domains (Frank & Brien, 2008). Discrimination and challenges in communication can make the FENs feel devalued and reduce their personal and professional confidence (Alexis et al., 2007). This in turn affects the way they interact with colleagues and patients and how they carry out their daily tasks. Teamwork is of the utmost importance for preventing patient safety risks and should receive special attention when employing FENs. One of the patient safety competencies is effective and appropriate participation on an interprofessional healthcare team, which requires a shared vocabulary to facilitate adequate communication in the team (Frank & Brien, 2008).

Lack of language proficiency affects FENs who work in a country with a very different language from their own, but can also apply to FENs who speak English in an English‐speaking country. Colloquial expressions, medical terminology, abbreviations and names of medication and equipment can differ in the new country (Jeon & Chenoweth, 2007). All these factors can create misunderstandings that threaten patient safety. The style of communication can also vary and FENs’ cultural adaptation to authority structures may prevent them from asking if they are in doubt about what to do. Requesting support when appropriate is another of the key safety competencies (Frank & Brien, 2008).

Habermann and Stagge (2010) assume that differences in education levels, language abilities and maladjustment to the cultural context can constitute a challenge to the safety of patients and the quality of care. Interaction in the staff room is especially important in multicultural groups as it fosters group cohesion and makes space for the host nurses and FENs to learn from each other (Xiao et al., 2014). A way of supporting FENs who feel alienated from their colleagues and lack a social network is described in a cultural education programme (Xiao et al., 2014). The authors explain that because of the lack of cultural sensitivity in nurse–nurse intercultural encounters in one hospital, they held a forum where they asked several immigrant nurses to tell their stories and describe the culture and practice in their home countries. The involvement of both host and immigrant nurses in the forum supported the two‐way approach of learning in multicultural settings. These learning outcomes will probably affect the patient safety culture, such as attitudes and values.

The cultural dissonance that FENs experience is related to both their work and their social life, which may affect their risk management. When FENs are subjected to discrimination or lack of support from their leaders or worry about their license, it can make them reluctant to disclose adverse events (Connor & Miller, 2014; Wheeler & Foster, 2013). The facilitation of continuity of care is of the utmost importance for patient safety. However, the results of this review revealed that it is a demanding area for new FENs.

### 5.2 Unfamiliar nursing practice

The theme “Unfamiliar nursing practice” is related to what the FENs do and how they practice nursing in a new environment. The two sub‐themes are “handling work‐related stress” and “role uncertainty and difficulties in decision‐making”. New nursing roles that emphasize a consumer‐centred approach and patient education were found to be unfamiliar to many FENs, while the demand for independent decision‐making led to uncertainty and indecision. If FENs do not ask questions or refrain from telling someone about their uncertainties, not only will teamwork and collaboration be affected, but the management of safety risks could be impaired. For example, in critical situations the proper handling and maintenance of equipment and the safe administration of medication require situational awareness. The safety competencies emphasize the integration of non‐technical skills with clinical knowledge and techniques as a necessary prerequisite to reduce the likelihood of harm (Frank & Brien, 2008). The FENs in our review expressed a wish for a longer period of nursing orientation and a need for hands‐on practical training and coaching to use their nursing knowledge and skills in a new system (Jose, 2011).

Newton, Pillay, and Higginbottom (2012), suggest that individual strategies for easing FENs’ transition to a new practice context could be taught through mentorship by experienced FENs and nurses from the host country. Røsnæs, Jølstad, Severinsson, and Lyberg (2017) found that reflection is a crucial part of nurse specialist students’ professional development towards ensuring patient safety and clinical supervision is an essential prerequisite for learning and acting in a reflective, professional manner (Jokelainen, Turunen, Tossavainen, Jamookeeah, & Coco, 2011; Woodbridge & Bland, 2010). As FENs come from a diverse range of educational and cultural backgrounds, reflective skills provide an opportunity to clarify their own values as well as learn some of the professional values in their new practice. Clinical supervision has been defined as a learning, supportive and monitoring process and therefore suitable for meeting these needs (Cutcliffe, Hyrkas, & Fowler, 2010; Woodbridge & Bland, 2010). Continual reflective activity will increase nurses’ self‐awareness and provide them with more options for dealing with unfamiliar patient situations and thinking critically about their own practice (Ekebergh, 2007).

Providing resources such as a mentor or supervisor who can help clarify certain information will facilitate FENs’ success in and satisfaction with their job performance (Reyes, 2013). However, Ginsburg et al. (2015) contend that internal reflection is not enough to foster self‐improvement. A learner also receives feedback from external sources. Both organizational structures and the work environment can therefore be linked to patient safety (Richardson & Storr, 2010). The importance of a supportive professional practice environment and organizational structures for integrating FENs should therefore be emphasized. Access to the necessary resources to do the job and having the opportunity to work and grow will empower employees to accomplish their duties in a meaningful way (Laschinger, 2008). For all nurses and especially FENs, system‐level interventions such as increasing nurse staffing and creating a better work environment have been associated with improved patient safety outcomes and a higher degree of nurse well‐being (Aiken et al., 2012; Bruyneel et al., 2013).

The full utilization of FENs knowledge and skills will depend to a large extent on their integration into the healthcare team (Allan, 2007; Buchan, 2006). The cultural diversity of the healthcare workforce poses a risk to patient safety. Quality and safety competencies may be interpreted differently across cultures and systems and these differences may challenge the safe integration of FENs into nursing practice (Sherwood & Shaffer, 2014). A challenge for many countries is ensuring that FENs receive equal treatment to nurses from the host country. It is important that healthcare institution managers are aware of the cultural and professionals needs of FENs and take appropriate action. When the FENs’ needs are met, they are better able to perform and provide safe, high quality patient care (Reyes, 2013). Victorino Beechinor and Fitzpatrick (2008) contend that cultural differences and workplace values are important factors to consider when recruiting a FEN. Nevertheless, integration can be problematic if it aims to remove differences between FENs and non‐migrant nurses by incorporating FENs into existing nursing systems (Raghuram, 2007). A two‐way approach where receiving healthcare institutions apply a culturally sensitive perspective seems to be of importance. Such a perspective should therefore be included in transition programmes for newly recruited FENs (Adeniran et al., 2008; Nease, 2009; Sherman & Eggenberger, 2008).

### 5.3 Methodological considerations

The strength of this study is the literature search and inclusion process, which involved developing a search strategy with a specialized librarian and the use of three reviewers. The first database search was conducted in 2014 and the second in 2016. Only one new article was found in the second search. However, we are aware that our choice of search terms and inclusion criteria may have affected the credibility. The review is limited to qualitative studies covering the FENs’ perspective. Quantitative studies and the perspectives of other healthcare professionals could have enriched the data, especially if presented to policy makers. An additional limitation is that the review only included articles written in the English language. When conducting a systematic review, data are decontextualized and removed from their original context, implying the risk that important findings in the primary research may be overlooked.

## 6 CONCLUSIONS

The fact that FENs perceive themselves as outsiders in their new country can affect their self‐confidence and professional nursing practice and pose a threat to patient safety. Nursing performance and skills are not universal, but linked to differences in culture and social norms. There is diversity in nursing practice and FENs should be supported to strengthen their patient safety competencies. A better prepared and longer orientation period and continual clinical supervision with systematic reflection on practice experiences is needed to support the transition of FENs to a new practice environment and to ensure patient safety. It is important that measures are taken both at individual and system level. Nurse managers are vital for ensuring the inclusion of FENs, involving them in decision‐making and providing appropriate working conditions, which will contribute to better quality of care and safeguard patients.

## CONFLICT OF INTEREST

The authors declare that there are no conflicts of interest.

## AUTHOR CONTRIBUTION

The study was designed by B.V., E.M.S and A.L. E.M.S. and A.L. participated in the data analysis and interpretation. The report was written by B.V. and A. L. All authors agreed on the final version and met at least one of the following criteria recommended by the ICMJE (http://www.icmje.org/recommondations/)

- substantial contributions to conception and design, acquisition of data or analysis and interpretation of data
- drafting the article or critically revising it for important intellectual content

## APPENDIX 1

### 1.1

**Table:** Table A1 Critical appraisal of the included studies (*n* = 17) by the three authors working independently with the CASP Qualitative Checklist criteria (CASP, 2013)

Author	1	2	3	4	5	6	7	8	9	10	Assessment
1 Almutairi et al. (2015)	Y	Y	Y	Y	Y	Y	Y	U	Y	Y	High
2 Connor and Miller (2014)	Y	Y	Y	Y	Y	N	Y	Y	Y	Y	High
3 Estacio and Saidy‐Khan (2014)	Y	Y	Y	Y	Y	Y	Y	U	Y	Y	Moderate
4 Kishi et al. (2014)	Y	Y	Y	Y	Y	Y	Y	Y	Y	Y	High
5 Xiao et al. (2014)	Y	Y	Y	Y	Y	U	Y	Y	Y	Y	High
6 Zhou (2014)	Y	Y	Y	Y	Y	Y	Y	Y	Y	Y	High
7 Alexis (2013)	Y	Y	Y	Y	Y	U	Y	Y	Y	Y	High
8 Lin (2013)	Y	Y	Y	Y	Y	Y	Y	U	Y	U	Moderate
9 Wheeler et al. (2013)	Y	Y	Y	Y	U	Y	Y	U	U	U	Moderate
10 Alexis and Shillingford (2011)	Y	Y	Y	Y	Y	U	Y	Y	Y	Y	High
11 Jose (2011)	Y	Y	Y	Y	Y	U	Y	Y	Y	Y	High
12 Liou and Cheng (2011)	Y	Y	Y	Y	Y	Y	Y	Y	Y	Y	High
13 Smith et al. (2011)											
14 Allan (2010)	Y	Y	Y	U	U	N	U	U	Y	U	Moderate
15 Tregunno et al. (2009)	Y	Y	Y	Y	Y	U	Y	U	Y	Y	Moderate
16 Xu et al. (2008)	Y	Y	Y	U	Y	Y	Y	Y	U	Y	High
17 Magnusdottir (2005)	Y	Y	Y	U	Y	U	U	U	Y	Y	Moderate

Y, Yes; U, Uncrtain; N, No.Screening questions: 1. Was there a clear statement of the aims of the research? 2. Is a qualitative methodology appropriate? 3. Was the research design appropriate to address the aim of the research? 4. Was the recruitment strategy appropriate to the aims of the research? 5. Were the data collected in a way that addressed the research issue? 6. Has the relationship between researcher and participants been adequately considered? 7. Have ethical issues been taken into consideration? 8. Was the data analysis sufficiently rigorous? 9. Is there a clear statement of findings? 10. How valuable is the research?
